# Effects of non-intubated spontaneous breathing on cerebral oxygen saturation and postoperative cognition in elderly patients with lung cancer undergoing video-assisted thoracoscopic surgery

**DOI:** 10.4103/mgr.MEDGASRES-D-25-00181

**Published:** 2025-10-02

**Authors:** Lei Zhang, Yuwen Lao, Yufan Zhang

**Affiliations:** 1 Department of Anesthesiology, Affiliated Jinhua Hospital, Zhejiang University School of Medicine: Jinhua Municipal Central Hospital, Jinhua, Zhejiang Province, China; 2 Department of Pain Treatment, Affiliated Jinhua Hospital, Zhejiang University School of Medicine: Jinhua Municipal Central Hospital, Jinhua, Zhejiang, China

**Keywords:** cerebral oxygen saturation, elderly, inflammation, stress, lung cancer, non-tracheal intubation anesthesia, postoperative cognitive dysfunction, preserved spontaneous breathing, risk factor, video-assisted thoracoscopic surgery

## Abstract

Video-assisted thoracoscopic surgery during one-lung ventilation may impair cerebral oxygen balance and increase the risk of postoperative cognitive impairment. This perspective study aimed to analyze the effect of non-intubated anesthesia with preserved spontaneous breathing on cerebral oxygen saturation and postoperative cognitive dysfunction in elderly patients with lung cancer during video-assisted thoracoscopic surgery. A total of 104 elderly patients with lung cancer who underwent video-assisted thoracoscopic surgery in Jinhua Municipal Central Hospital from January 2023 to October 2024 were selected, and they were randomly divided into non-intubated group (*n* = 52) and intubated group (*n* = 52). The cerebral oxygen saturation, postoperative cognitive dysfunction incidence and laboratory indicators were compared between the two groups. At last, the influencing factors to postoperative cognitive dysfunction were analyzed by multivariate Logistic regression. The cerebral oxygen saturation was higher at 15, 30 and 60 minutes after operation than at the beginning of anesthesia, and moreover, it in the non-intubation group were significantly higher than that in the intubated group (*P* < 0.05). At 72 hours after operation, the levels of tumor necrosis factor-α, interleukin-6 and S100β in serum were lower than those before operation, and the levels in the non-intubated group were significantly lower than those in the intubated group; the levels of epinephrine, atrial natriuretic peptide and cortisol in serum were higher than those before operation, and they in the non-intubated group were significantly more than those in the intubated group (*P* < 0.05). The total incidence of postoperative cognitive dysfunction in intubation group was higher than that in the non-intubated group within 2 months after operation (*P* < 0.05). Multivariate Logistic regression analysis showed that age (odds ratio = 1.729), cerebral oxygen saturation at 30 minutes after operation (odds ratio = 0.727) and cerebral oxygen saturation at 60 minutes after operation (odds ratio = 0.734) were independent influencing factors for postoperative cognitive dysfunction (all *P* < 0.05). Non-tracheal intubation anesthesia with preserved spontaneous breathing can increase cerebral oxygen saturation, reduce systemic inflammatory response and stress response, and decrease the incidence of postoperative cognitive dysfunction in elderly patients with lung cancer undergoing video-assisted thoracoscopic surgery, which is conducive to promoting postoperative recovery in such patients.

## Graphical abstract

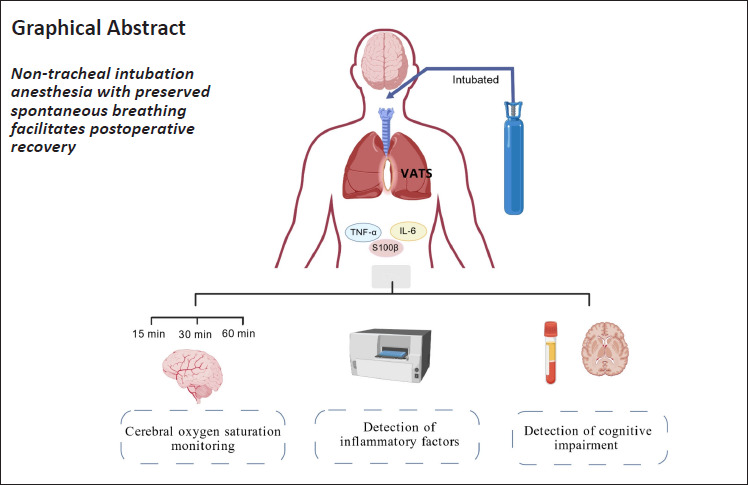

## Introduction

Previous studies suggest that in lung cancer treatment, video-assisted thoracoscopic surgery (VATS) during unilateral lung ventilation may compromise cerebral oxygen balance^1^ and increase the risk of postoperative cognitive dysfunction (POCD),^2^ severely affecting patients’ postoperative recovery and quality of life, while imposing additional burdens on society and families.^3^,^4^ To reduce the ventilatory impact caused by tracheal intubation during VATS, it is necessary to select more appropriate ventilation methods to minimize damage.^5^ Studies have found that patients receiving non-intubated anesthesia can increase intraoperative carbon dioxide partial pressure concentration, leading to elevated regional cerebral oxygen saturation and reduced POCD rate.^6^,^7^,^8^ During VATS procedures in elderly lung cancer patients, artificial pneumothorax and unilateral lung ventilation result in pulmonary compression and mediastinal shift, thereby reducing the ventilation-perfusion ratio of the contralateral lung.^9^,^10^,^11^ Consequently, respiratory frequency or tidal volume must be increased to meet gas exchange requirements. However, few studies have yet explored the efficacy of non-tracheal intubation anesthesia with preserved spontaneous breathing in elderly lung cancer patients undergoing VATS. Therefore, this perspective study aimed to analyze the effects of non-tracheal intubation anesthesia with preserved spontaneous breathing on cerebral oxygen saturation and the occurrence of POCD.

## Methods

### Subjects

After approval by the Ethics Committee of Jinhua Municipal Central Hospital [No. (Research) 2022840101], 104 elderly patients with lung cancer who underwent VATS surgery at Jinhua Municipal Central Hospital from January 2023 to October 2024 were selected as study subjects. Patients and their legal guardians were informed about the study and signed informed consent forms. The study protocol was conformed to the principles outlined in the Declaration of Helsinki. The patients met the following inclusion criteria: (1) Histopathologically confirmed non-small cell lung cancer^12^ meeting the indications for radical surgery; (2) Age ≥ 60 years; (3) American Society of Anesthesiologists Standard classification^13^ grade I-II, and tumor-node-metastasis staging^14^ I–II; (4) Mini-Mental State Examination score^15^ ≥ 24 points. Patients who meet the following criteria are excluded from the trial: (1) Patients with severe chronic obstructive pulmonary disease, pulmonary infection, or extensive pleural adhesions; (2) Patients with preoperative hypoxemia, hypercapnia, or other complications; (3) Patients with hematological or immunological diseases; (4) Patients with contraindications to paravertebral nerve block and intercostal nerve block; (5) Patients with severe hepatic or renal dysfunction; (6) Patients with difficult airway management; (7) Patients with changes in surgical plan or anesthetic method; (8) Patients requiring other treatments due to postoperative deterioration or hospital transfer.

**Figure 1:**
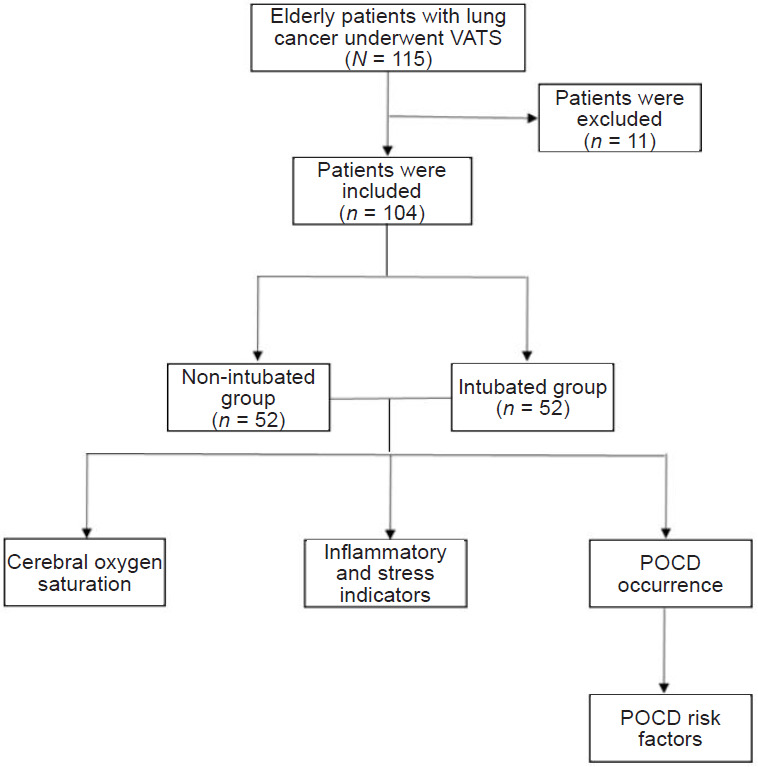
Study design. POCD: Postoperative cognitive dysfunction; VATS: video-assisted thoracoscopic surgery.

Using a random number table method, enrolled patients were divided into a non-intubated group (*n* = 52) and an intubated group (*n* = 52). General data, including sex, age, body mass index, smoking history, drinking history, education level, American Society of Anesthesiologists standard classification, tumor–node–metastasis staging, Mini-Mental State Examination score, pathological types and tumor location, were collected. The study design was detailed in **Figure 1**.

### Anesthesia

The two groups received identical preoperative management and anesthetic monitoring. All patients fasted for 8 hours and abstained from water for 4 hours prior to surgery. Peripheral venous access was established, and heart rate, pulse, and respiratory rate were monitored. VATS was performed under ultrasound guidance.

Non-intubated group received anesthetic induction consisting of midazolam (0.05 mg/kg intravenous injection; Jiangsu Enhua Pharmaceutical, Xuzhou, Jiangsu Province, China) + dexamethasone (10 mg intravenous injection; Baizheng Pharmaceutical, Xuchang, Henan Province, China) + sufentanil (0.1 μg/kg intravenous injection; Jiangsu Enhua Pharmaceutical) + etomidate (0.2–0.4 mg/kg intravenous injection; Jiangsu Enhua Pharmaceutical). When the bispectral index decreased to 60, an appropriately sized laryngeal mask was inserted with oxygen flow rate set at 2–3 L/min and oxygen concentration at 50–100%. Blood pressure fluctuations were controlled within 20% of baseline values. Anesthesia maintenance infused with micro-pump included propofol (2–3 mg/kg/h; Sichuan Guorui Pharmaceutical, Leshan, Sichuan Province, China) + remifentanil (Jiangsu Enhua Pharmaceutical) 0.03–0.08 μg/kg/min + dexmedetomidine (0.3–1 μg/kg/h; Sichuan Guorui Pharmaceutical) + flurbiprofen axetil (50 mg; Guangdong Jiabo Pharmaceutical, Qingyuan, Guangdong Province, China). Dosages were adjusted according to the bispectral index to maintain levels between 40 and 60. Local anesthesia was performed using 1% lidocaine (Beijing Yimin Pharmaceutical, Beijing, China) 5 mL around skin incision. Under thoracoscopic guidance, intercostal nerve blocks were performed using a 1:1 mixture of 1% ropivacaine (Jichuan Pharmaceutical, Taixing, Jiangsu Province, China) and 2% lidocaine, with 1 mL injected at each intercostal nerve position innervating surgical incision and drainage tube. Pleural cavity anesthesia was achieved by injecting 2% lidocaine 5 mL into the space between parietal and visceral pleura. Intrathoracic vagal nerve block was performed using 2.5 mL of a 1:1 mixture of 1% ropivacaine and 2% lidocaine. During anesthesia, if oxygen saturation fell below 90% or end-tidal CO_2_ pressure continuously increased above 80 mmHg, the ventilation mode should be adjusted to synchronized intermittent mandatory ventilation or manual ventilation to improve ventilation quality.

Intubated group received anesthetic induction including midazolam (0.05 mg/kg) + sufentanil (0.5 μg/kg) + dexamethasone (10 mg) + etomidate (0.2–0.4 mg/kg) + rocuronium (1 mg/kg; Zhejiang Xianju Pharmaceutical, Taizhou, Zhejiang Province, China). When the bispectral index decreased to 60, a double-lumen endotracheal tube was inserted for mechanical ventilation with mask pressurized oxygen flow rate set at 2–3 L/min and oxygen concentration at 50–100%. Blood pressure fluctuations were controlled within 20% of baseline values. Anesthesia maintenance included propofol 4–12 mg/kg/h + remifentanil 0.03–0.08 μg/kg/min + dexmedetomidine 0.3–1 μg/kg/h + intravenous flurbiprofen axetil 50 mg. Dosages were adjusted according to the bispectral index to maintain levels between 40 and 60.

All patients were returned to the ward after complete awakening and vital sign assessment. Postoperative analgesia was provided using patient-controlled intravenous analgesia pumps (sufentanil 1 μg/mL mixed with 0.9% normal saline to 100 mL) with a loading dose of 2 mL, infusion rate of 2 mL/h, patient-controlled bolus dose of 3 mL, and lockout time of 30 minutes. Postoperative pain was assessed using the Visual Analogue Scale.^16^ If pain scores persistently exceeded 5 or patients experienced unbearable pain, intravenous flurbiprofen axetil 50 mg was administered.

### Outcome measures

#### Cerebral oxygen saturation

Using a cerebral tissue oxygen saturation monitor (Wuhan Bolianzhongke Technology Co., Ltd., Wuhan, China, MOC200, Device Registration No. 20212073187), cerebral oxygen saturation levels were measured preoperatively, at anesthesia induction, and 15, 30 and 60 minutes after surgery initiation.

#### Inflammatory and stress responses

Cubital venous blood samples (5 mL) were collected preoperatively and 72 hours postoperatively. Enzyme-linked immunosorbent assay (Sigma, St. Louis, MO, USA) was used to detect the levels of tumor necrosis factor-α, interleukin-6, S100β, epinephrine, atrial natriuretic peptide, and cortisol in serum.

#### Occurrence of postoperative cognitive dysfunction

The occurrence of POCD was evaluated according to diagnostic criteria,^17^,^18^ including corresponding clinical symptoms (such as memory loss, decreased executive function, lack of concentration, impaired language function, etc.), which should last for several weeks to months. The Mini-Mental State Examination score decrease by ≥ 2 points after surgery compared to before, and other diseases, such as dementia, depression, metabolic disorders, should be excluded.

#### Risk factors for postoperative cognitive dysfunction

We divided the 104 patients into two groups based on whether POCD occurred or not, they were cognitive dysfunction group and non-cognitive dysfunction group. The univariate comparison of data between the two groups including demographic information, tumor-node-metastasis staging, Mini-Mental State Examination score, anesthesia duration, operation time, cerebral oxygen saturation, S100β, epinephrine, atrial natriuretic peptide, cortisol and interleukin-6 levels, and lung function indicators (forced vital capacity and forced expiratory volume in the first second), were performed, and then multivariate regression analysis was used to screen for risk factors.

### Sample size calculation

Referring to previous literature reports, the incidence of POCD in lung cancer was 20–30%,^19^,^20^ after using autonomous breathing anesthesia, the incidence of POCD was reduced to at least 10–15%. The alpha and beta values were taken as 0.05 and 0.80, respectively. The calculated sample size was 105 cases. Adding at least 5–10% sample size loss, the total number of patients included was 115.

### Statistical analysis

Data processing was performed using SPSS 25.0 statistical software (IBM, Armonk, NY, USA). Categorical data were expressed as number (percentage), with unordered dichotomous or multicategorical data analyzed using Chi-square test and ordinal data analyzed using rank-sum test. Continuous data were expressed as mean ± standard deviation and analyzed using independent-sample *t*-test, or repeated measures analysis of variance followed by least significant difference test. Multivariate analysis employed logistic regression. *P* < 0.05 indicated statistical significance.

## Results

### Comparison of general data between intubated and non-intubated groups

According to the inclusion and exclusion criteria, 104 patients were included in the experiment. The general data between the two groups showed no statistically significant differences (*P* > 0.05; **Table 1**).

**Table 1:** Comparison of general data between intubated and non-intubated groups

	Intubated group (*n* = 52)	Non-intubated group (*n* = 52)	*χ*²/*Z*/*t*	*P*
Age (yr)	70.43±5.37	69.84±6.71	0.495	0.622
Sex			0.991	0.319
Male	24(46)	19(37)		
Female	28(54)	33(63)		
BMI (kg/m^2^)	22.10±2.48	22.69±2.52	1.203	0.232
Smoking	15(29)	13(25)	0.195	0.658
Drinking	12(23)	18(35)	1.686	0.194
ASA classification			1.182	0.277
I	6(12)	10(19)		
II	46(88)	42(81)		
TNM staging			0.619	0.431
I	30(58)	26(50)		
II	22(42)	26(50)		
Education level			0.892	0.64
Junior and below	5(10)	3(6)		
Senior	25(48)	29(56)		
College and above	22(42)	20(38)		
MMSE score	28.94±1.90	29.15±2.33	0.504	0.616
Pathological type			1.195	0.274
Squamous cell carcinoma	35(67)	40(77)		
Adenocarcinoma	17(33)	12(23)		
Tumor location			1.149	0.284
Peripheral type	39(75)	34(65)		
Central type	13(25)	16(35)		

Data in age, BMI and MMSE score are expressed as mean ± SD, and were analyzed by independent-sample *t*-test. Data in sex, ASA classification, TNM staging, education level, pathological type and tumor location are expressed as number (percentage), and were analyzed by Chi-square analysis. Data in smoking, and drinking are expressed as number (percentage), and were analyzed by *Z*-test. ASA: American Society of Anesthesiologists; BMI: body mass index; MMSE: Mini-Mental State Examination; TNM: tumor–node–metastasis.

### Comparison of cerebral oxygen saturation levels in elderly lung cancer patients between intubated and non-intubated groups

A repeated measures analysis of variance showed significant differences in cerebral oxygen saturation between groups (*F* = 58.104, *P* = 0.000) and among groups (*F* = 18.578, *P* = 0.000), but there was no difference in the interaction (*F* = 1.571, *P* = 0.124). There were no statistically significant differences in cerebral oxygen saturation levels between the two groups preoperatively and at anesthesia induction (*P* > 0.05). Cerebral oxygen saturation at 15, 30, and 60 minutes after surgery were significantly higher than those at anesthesia induction in the two groups (*P* < 0.05), what’s more, they in the non-intubated group were significantly higher than those in the intubated group (*P* < 0.05; **Table 2**).

**Table 2:** Comparison of cerebral oxygen saturation levels (mmHg) in elderly lung cancer patients between intubated and non-intubated groups

	Preoperative	At anesthesia induction	15 min after surgery	30 min after surgery	60 min after surgery
Intubated group (*n* = 52)	70.16±6.52	72.68±4.48*	72.69±3.46*	72.12±3.16*	70.03±4.11
Non-incubated group (*n* = 52)	70.34±7.19	72.44±3.72*	76.94±4.03*	77.06±3.84*	75.19±3.67*

*t*	0.134	0.297	5.77	7.163	6.753
*P*	0.894	0.767	0	0	0

Data are expressed as mean ± SD, and were analyzed by analyzed by independent-sample *t*-test. *P < 0.05, *vs*. preoperative.

### Comparison of inflammatory and stress indicators in elderly lung cancer patients between intubated and non-intubated groups

There were no significant differences in preoperative tumor necrosis factor-α, interleukin-6, S100β, epinephrine, atrial natriuretic peptide, and cortisol levels between the two groups (*P* > 0.05). At 72 hours postoperatively, tumor necrosis factor-α, interleukin-6, and S100β levels were lower than preoperative levels in both groups, with the non-intubated group showing lower levels than the intubated group. Epinephrine, atrial natriuretic peptide, and cortisol levels were higher than preoperative in the two groups, with the intubated group showing higher levels than the non-intubated group (*P* < 0.05; **Table 3**).

**Table 3:** Comparison of inflammatory and stress indicators in elderly lung cancer patients between intubated and non-intubated groups

Indicators	Time	Intubated group (*n* = 52)	Non-intubated group (*n* = 52)	*t*	*P*
TNF-α (fg/mL)	Preoperative	460.38±100.72	465.65±132.09	0.229	0.820
	72 h postoperative	443.06±97.68	410.16±84.39*	3.838	0.001
IL-6 (pg/mL)	Preoperative	98.48±21.16	99.52±19.24	0.262	0.794
	72 h postoperative	85.06±13.91*	75.22±14.08*	3.585	0.001
S100β (pg/mL)	Preoperative	729.08±182.85	715.20±167.77	0.403	0.688
	72 h postoperative	695.69±141.25	631.53±128.04*	2.427	0.017
E (pg/mL)	Preoperative	33.49±6.07	33.17±5.82	0.274	0.784
	72 h postoperative	48.39±5.44*	37.62±5.71*	9.848	0.000
ANP (nM)	Preoperative	0.23±0.06	0.22±0.08	0.721	0.473
	72 h postoperative	0.32±0.09*	0.27±0.07*	3.162	0.002
Cor (μg/dL)	Preoperative	10.19±2.06	10.31±2.14	0.291	0.771
	72 h postoperative	19.26±3.40*	11.26±2.38*	13.900	0.000

Data are expressed as mean ± SD, and were analyzed by analyzed by independent-sample *t*-test. *P < 0.05, *vs*. preoperative. ANP: Atrial natriuretic peptide; Cor: cortisol; E: epinephrine; IL-6: interleukin-6; S100β: a central nervous system specific protein; TNF-α: tumor necrosis factor-α.

### Comparison of cognitive dysfunction occurrence in elderly lung cancer patients between intubated and non-intubated groups

The total incidence of cognitive dysfunction within 2 months postoperatively was higher in the intubated group than in the non-intubated group (*P* < 0.05; **Table 4**).

**Table 4:** Comparison of cognitive dysfunction occurrence (%) between intubated and non-intubated groups in elderly lung cancer patients

Group	7 d postoperative	14 d postoperative	30 d postoperative	2 mon postoperative	Total incidence
Incubated group (*n* = 52)	7(13)	4(8)	2(4)	1(2)	14(27)
Non-incubated group (*n* = 52)	3(6)	1(2)	0	0	4(8)

*χ* ^2^	1.770	1.891	2.039	1.010	6.718
*P*	0.996	0.169	0.153	0.315	0.010

Data in are expressed as number (percentage), and were analyzed by Chi-square analysis.

### Comparison of general characteristics between elderly lung cancer patients with and without cognitive dysfunction

Significant differences were observed between the cognitive dysfunction group and the non-cognitive dysfunction group in age, anesthesia duration, S100β levels, interleukin-6 levels, cerebral oxygen saturation at 30 and 60 minutes after surgery initiation, and forced vital capacity (*P* < 0.05; **Table 5**).

**Table 5:** Comparison of general characteristics between elderly lung cancer patients with and without cognitive dysfunction

	Cognitive dysfunction group (*n* = 18)	Non-cognitive dysfunction group (*n* = 86)	*χ*²/*Z*/*t*	*P*
Age (yr)	73.69±4.87	67.34±4.14	5.156	0.000
Sex			1.812	0.178
Male	10(56)	33(38)		
Female	8(44)	43(62)		
BMI (kg/m^2^)	22.10±2.48	22.69±2.52	0.915	0.369
Smoking	8(44)	20(23)	3.397	0.065
Drinking	6(33)	24(28)	0.214	0.644
ASA classfication			2.568	0.109
I	5(28)	11(13)		
II	13(72)	75(87)		
TNM staging			0.774	0.379
I	8(44)	48(56)		
II	10(56)	38(44)		
Education level			5.090	0.078
Junior and below	2(11)	6(7)		
Senior	13(72)	41(48)		
College and above	3(17)	39(45)		
MMSE score	28.94±1.90	29.15±2.33	0.409	0.686
Anesthesia duration (min)	130.22±23.65	119.26±21.71	1.813	0.083
Operation time (min)	118.40±20.89	103.62±19.76	2.755	0.011
S100β (pg/mL)	799.49±149.42	702.35±109.84	2.614	0.016
IL-6 (pg/mL)	109.95±22.16	96.12±20.57	2.437	0.023
TNF-α (fg/mL)	475.27±112.34	468.25±105.92	1.032	0.236
E (pg/mL)	35.67±5.96	32.59±5.42	0.859	0.302
ANP (nM)	0.24±0.03	0.23±0.03	0.657	0.421
Cor (μg/dL)	12.23±2.63	10.89±2.41	0.895	0.369
Cerebral oxygen saturation (%)				
Preoperative	70.26±7.11	71.05±6.84	0.431	0.670
30 min after surgery	70.41±6.95	80.59±7.11	5.628	0.000
60 min after surgery	69.16±5.33	76.59±6.51	5.163	0.000
FVC (%)	76.15±10.42	86.22±11.16	3.682	0.001
FEV1 (%)	86.63±11.03	87.39±10.01	0.270	0.790

Data in age, BMI, MMSE score, anesthesia duration, operation time, S100β, IL-6, TNF-α, E, ANP, Cor, cerebral oxygen saturation, FVC, and FEV1 are expressed as mean ± SD, and were analyzed by independent-sample *t*-test. Data in sex, ASA classification, TNM staging, education level, pathological type and tumor location are expressed as number (percentage), and were analyzed by Chi-square analysis. Data in smoking, and drinking are expressed as number (percentage), and were analyzed by *Z*-test. ANP: Atrial natriuretic peptide; ASA: American Society of Anesthesiologists; BMI: body mass index; Cor: cortisol; E: epinephrine; FEV1: forced expiratory volume in the first second; FVC: forced vital capacity; IL-6: interleukin-6; MMSE: Mini-Mental State Examination; S100β: a central nervous system specific protein; TNF-α: tumor necrosis factor-α; TNM: tumor–node–metastasis.

### Multivariate analysis of postoperative cognitive dysfunction in elderly lung cancer patients with video-assisted thoracoscopic surgery

Multivariate logistic regression analysis revealed that age (odds risk = 1.729, 95% confidence interval = 1.019–2.935, *P* = 0.042), cerebral oxygen saturation at 30 (odds ratio = 0.727, 95% confidence interval = 0.575–0.918, *P* = 0.007), and 60 minutes after surgery initiation (odds ratio = 0.734, 95% confidence interval = 0.545–0.989, *P* = 0.042) were independent risk factors for POCD in elderly lung cancer VATS patients (all P < 0.05; **Table 6**).

**Table 6:** Multivariate logistic regression analysis of postoperative cognitive dysfunction in elderly lung cancer patients with video-assisted thoracoscopic surgery (VATS)

Variables	β	Standard error	*Wald*	*P*	*OR*	95% confidence interval	
						Lower limit	Upper limit
Age	0.548	0.27	4.119	0.042	1.729	1.019	2.935
Anesthesia duration	0.019	0.029	0.442	0.506	1.019	0.964	1.078
S100β	0.047	0.068	0.481	0.488	1.048	0.917	1.199
IL-6	–0.011	0.059	0.032	0.858	0.99	0.882	1.11
Cerebral oxygen saturation at 30 min after surgery initiation	–0.319	0.119	7.184	0.007	0.727	0.575	0.918
Cerebral oxygen saturation at 60 min after surgery initiation	–0.309	0.152	4.13	0.042	0.734	0.545	0.989
FVC	–0.122	0.067	3.34	0.068	0.885	0.776	1.009

FVC: Forced vital capacity; IL-6: interleukin-6; OR: odds ratio; S100β: a central nervous system specific protein.

## Discussion

During anesthesia, anesthetic agents reduce pulmonary respiratory frequency or tidal volume. In the study by Prisciandaro et al.^21^ non-intubated ventilation was more likely to cause inadequate spontaneous ventilation compared to intubated ventilation, manifesting as elevated cerebral oxygen saturation levels. Our study also found that patients in the non-intubated group maintained higher and more stable cerebral oxygen saturation levels than the intubated group from 15 minutes after surgery initiation, indicating that non-tracheal intubation anesthesia with spontaneous breathing can increase intraoperative regional cerebral oxygen saturation. Furthermore, comparison of inflammatory and stress response indicators between the two groups before and after surgery revealed that tumor necrosis factor-α, interleukin-6, and S100β levels were all lower at 72 hours postoperatively than preoperative levels, with the non-intubated group showing lower levels than the intubated group. Conversely, epinephrine, atrial natriuretic peptide, and cortisol levels were higher than preoperative levels, with the intubated group showing higher levels than the non-intubated group. These findings demonstrate that non-tracheal intubation anesthesia with spontaneous breathing offers advantages in reducing postoperative inflammatory and stress responses in elderly lung cancer VATS patients. Chen et al.^22^ suggested that non-intubated anesthesia avoids tracheal and pharyngeal stimulation from intubation, preventing excessive stress responses caused by external stimulation and stabilizing the internal environment. Additionally, the elevation of cerebral oxygen saturation achieved through non-tracheal intubation anesthesia with spontaneous breathing prevents central nervous system inflammatory responses activated by cerebral tissue hypoxia.^23^,^24^,^25^

POCD is a common central nervous system complication following surgical procedures, characterized by cognitive abnormalities involving impaired personality, social abilities, memory, attention, and comprehension.^26^,^27^ The peak incidence of POCD usually occurs 2 months after surgery. In this study, the overall incidence of cognitive dysfunction within 2 months postoperatively was higher in the intubated group than in the non-intubated group, indicating that non-tracheal intubation anesthesia with preserved spontaneous breathing can reduce the incidence of cognitive dysfunction in elderly lung cancer VATS patients. We believe this outcome may be related to cerebral oxygen saturation and inflammatory response levels. Huang et al.^26^ found that POCD occurrence was associated with hypercapnia, and the resulting elevation in cerebral oxygen saturation can stimulate cardiac sympathetic nerve excitation, enhance myocardial contractility and cardiac output, ultimately improving cerebral blood flow perfusion while preventing neuronal hypoxia and reducing neurological damage. Furthermore, appropriate elevation of cerebral oxygen saturation can prevent activation of inflammatory cytokines and increased oxidative stress levels caused by reduced cerebral blood flow perfusion, further protecting cerebral neuronal cell viability.^28^,^29^ Cai et al.^30^ similarly highlighted the positive effects of non-intubated ventilation on VATS surgical outcomes. Moreover, we found that POCD mainly occurred within 14 days after surgery, which was consistent with previous reports.^31^,^32^ Of course, the sample size of the two groups was small, which may lead to the low positive rate of POCD. A larger sample size may provide more stable and accurate results.

Through multivariate logistic regression analysis, this study identified age, cerebral oxygen saturation at 30 minutes after surgery initiation, and cerebral oxygen saturation at 60 minutes after surgery initiation as independent risk factors for POCD in elderly lung cancer VATS patients. During anesthesia, vasodilation and blood pressure reduction lead to decreased cerebral blood flow, while surgical stimulation and trauma cause inflammatory responses and elevated stress levels, increasing cerebral neuronal oxygen demand. This demand conflicts with reduced cerebral blood flow under anesthesia, ultimately resulting in decreased cerebral oxygen supply, relative hypoxia of cerebral neurons, and eventual cognitive dysfunction.^33^,^34^,^35^ Cerebral oxygen saturation reflects the balance between cerebral oxygen supply and demand; therefore, evaluating patients’ intraoperative cerebral oxygen saturation levels has predictive value for POCD occurrence. Ding et al.^34^ also found that abnormally decreased cerebral oxygen saturation levels were risk factors for neurocognitive dysfunction, consistent with our findings. Therefore, close monitoring of cerebral oxygen saturation levels in elderly lung cancer patients during surgery is essential to prevent cerebral neuronal tissue hypoxia and POCD caused by abnormal decreases in cerebral oxygen saturation during VATS procedures. Additionally, Moller et al.^36^ found that cognitive dysfunction is related to physiological degeneration of neurological function, with older patients having poorer physical functional status and being more susceptible to stimulation of neuronal cells by factors such as anesthesia and surgical trauma. Non-tracheal intubation anesthesia with reserved spontaneous breathing causes minimal tracheal stimulation, reducing patient stress responses to some extent and providing better stabilization of the internal environment in elderly lung cancer VATS patients, further reducing POCD occurrence.

The limitations of this study include a relatively small sample size and single-center design, which restricts the generalizability of findings. Future multicenter studies with larger sample sizes are needed to further validate these conclusions.

In conclusion, non-tracheal intubation anesthesia with preserved spontaneous breathing increases intraoperative cerebral oxygen saturation, helps reduce systemic inflammatory and stress responses, and does not increase POCD in elderly lung cancer VATS patients, thereby promoting postoperative recovery.

## Data Availability

*Not applicable.*
